# Contezolid can replace linezolid in a novel combination with bedaquiline and pretomanid in a murine model of tuberculosis

**DOI:** 10.1128/aac.00789-23

**Published:** 2023-11-15

**Authors:** Deepak Almeida, Si-Yang Li, Jin Lee, Barry Hafkin, Khisimuzi Mdluli, Nader Fotouhi, Eric L. Nuermberger

**Affiliations:** 1 Department of Medicine, Johns Hopkins University Center for Tuberculosis Research, Baltimore, Maryland, USA; 2 MicuRx Pharmaceuticals, Foster City, California, USA; 3 Global Alliance for Tuberculosis Drug Development, New York, USA; Houston Methodist Academic Institute, Houston, Texas, USA

**Keywords:** contezolid, *Mycobacterium tuberculosis*, oxazolidinones

## Abstract

Contezolid is a new oxazolidinone with *in vitro* and *in vivo* activity against *Mycobacterium tuberculosis* comparable to that of linezolid. Pre-clinical and clinical safety studies suggest it may be less toxic than linezolid, making contezolid a potential candidate to replace linezolid in the treatment of drug-resistant tuberculosis. We evaluated the dose-ranging activity of contezolid, alone and in combination with bedaquiline and pretomanid, and compared it with linezolid at similar doses, in an established BALB/c mouse model of tuberculosis. Contezolid had an MIC of 1 µg/mL, similar to linezolid, and exhibited similar bactericidal activity in mice. Contezolid-resistant mutants selected *in vitro* had 32- to 64-fold increases in contezolid MIC and harbored mutations in the *mce3R* gene. These mutants did not display cross-resistance to linezolid. Our results indicate that contezolid has the potential to replace linezolid in regimens containing bedaquiline and pretomanid and likely other regimens.

## INTRODUCTION

Despite recent advances in the development of new drugs against tuberculosis (TB), major challenges exist for the treatment of rifampin-resistant (RR-) TB. Linezolid (LZD), the first oxazolidinone antibacterial drug to reach the market, has been repurposed to treat RR-TB and is now designated as a preferred drug for this indication by the WHO (1). LZD exhibited additive bactericidal and sterilizing activities when combined with bedaquiline (BDQ) and pretomanid (PMD) in mouse models of TB (2, 3). The resulting 3-drug “BPaL” regimen later proved to be an effective 6-month, oral regimen for extensively drug-resistant and refractory multidrug-resistant TB and received FDA approval for this indication. Despite the efficacy of the BDQ-PMD-LZD regimen, the dose- and duration-dependent toxicity of LZD compromises its utility (4–6). Replacing LZD with newer oxazolidinones with similar activity and superior safety could reduce the burden of safety monitoring for treatment programs and expand the regimen’s spectrum of use to include drug-susceptible TB.

Contezolid (CZD), previously called MRX-I, is a newer oxazolidinone with potent activity against methicillin-, penicillin-, and vancomycin-resistant bacteria similar or superior to that of LZD (7). It was recently approved by the National Medical Products Administration in China for the treatment of complicated skin and soft tissue infections; it has already been used off-label in TB treatment (8). Although the MIC range of CZD against *Mycobacterium tuberculosis* (0.25–1.0 µg/mL) is similar to that of LZD (0.25–1.0 µg/mL), it may have a superior safety profile (7, 9, 10).

Here, we tested whether CZD could replace LZD in the BDQ-PMD-LZD regimen without loss of efficacy in a well-established mouse model of TB. CZD was used in a dose-ranging fashion, alone and in combination with BDQ-PMD, to evaluate its bactericidal activity relative to that of LZD. We also selected and characterized CZD-resistant mutants *in vitro* and evaluated them for cross-resistance with LZD.

## RESULTS

### MIC determination

MICs of CZD and LZD against the parent *M. tuberculosis* H37Rv strain were determined using the broth macrodilution method and found to be 0.5–1 and 0.5–1 µg/mL, respectively.

### Efficacy in mouse model of TB

The dose-ranging activity of CZD and LZD, both alone and in combination with BDQ and PMD, was evaluated in a subacute BALB/c mouse infection model. The results of lung colony-forming unit (CFU) counts are shown in Table 1. After 4 weeks of treatment, dose-dependent activity was observed for both oxazolidinones. Although the bacterial load did not increase as much as is typically observed in this model between infection and the initiation of treatment, the LZD effects observed in the active control arms were consistent with prior studies (2, 11). Mice in the LZD 100 mg/kg and CZD 100 and 200 mg/kg monotherapy groups had significantly lower mean CFU counts compared to pre-treatment (D0) controls (*P* < 0.05 for CZD at 100 mg/kg; *P* ≤ 0.0001 for others), while LZD and CZD at 50 mg/kg were largely bacteriostatic. The addition of each oxazolidinone increased the bactericidal activity of BDQ-PMD, although only LZD at 100 mg/kg and CZD at 100 or 200 mg/kg increased the activity of the BDQ-PMD combination in a statistically significant fashion (*P* < 0.05 for CZD at 100 mg/kg; *P* < 0.001 for others). BDQ-PMD plus CZD at 200 mg/kg produced the lowest mean CFU count but was not statistically superior to BDQ-PMD-LZD_100_.

**TABLE 1:** Mean lung CFU counts observed in the mouse efficacy experiment

	Mean (± SD) log_10_ CFU/lung		
Regimen^*a*^	Day 13	Day 0	Week 4
Untreated	3.59 ± 0.07	6.21 ± 0.18	
LZD_50_			6.09 ± 0.23
LZD_100_			5.37 ± 0.16
CZD_50_			6.15 ± 0.22
CZD_100_			5.78 ± 0.31
CZD_200_			5.36 ± 0.25
BDQ-PMD			1.39 ± 0.12
BDQ-PMD-LZD_50_			0.96 ± 0.16
BDQ-PMD-LZD_100_			0.52 ± 0.20
BDQ-PMD-CZD_50_			1.16 ± 0.44
BDQ-PMD-CZD_100_			0.81 ± 0.32
BDQ-PMD-CZD_200_			0.42 ± 0.32^*b*^

^*a*^ Subscripts indicate the LZD or CZD dose in mg/kg body weight

^*b*^ One of the five mice had no detectable CFU, the limit of detection was 1 CFU.

### Selection and characterization of drug-resistant mutants

To evaluate the potential for cross-resistance between CZD and LZD, mutants resistant to both drugs were selected *in vitro* and whole-genome sequencing was performed to identify mutations. The calculated frequency of resistance was approximately 2 × 10^−5^ and 4 × 10^−6^ at 3 and 10 µg/mL of CZD, respectively, compared to 3 × 10^−8^ for LZD. Each of the resistant colonies sequenced from the CZD-containing plates (1 from each CZD concentration) had acquired a stop codon mutation in the *mce3R* gene, affecting either the Tyr185 or the Tyr118 residue. No other new mutations were found to be in common between the resistant isolates. These mutants exhibited elevated CZD MICs of 32–64 µg/mL but retained full susceptibility to LZD (Table 2). By contrast, mutants selected on LZD-containing plates harbored either *rplC* (nt460 T→C; C154R) or *rrl* (nt2814 G→T) mutations. Both of these LZD-resistant mutants showed cross-resistance to CZD.

**TABLE 2:** Linezolid and contezolid MICs against the *M. tuberculosis* H37Rv parent strain and resistant mutants selected *in vitro*

Strain	LZD MIC (µg/mL)^*a*^	CZD MIC (µg/mL)
1) H37Rv parent	0.5–1	0.5–1
2) *mce3R* mutant selected on CZD 3 µg/mL	1	32
3) *mce3R* mutant selected on CZD 10 µg/mL	1	64
4) *rplC* C154R mutant selected on LZD	16–32	16
5) *rrl*nt2814 G→T mutant selected on LZD	16–32	32

^*a*^ LZD MICs against strains 1, 4, and 5 and CZD MIC against strain 1 were tested twice and the MIC range is shown.

## DISCUSSION

Short-course oral regimens based on the BDQ-PMD-LZD combination have demonstrated high rates of successful clinical outcomes in the treatment of RR-TB, but dose- and duration-dependent toxicity of LZD remains a challenge (4–6). There is great interest in identifying a safer and equally efficacious oxazolidinone to replace LZD. CZD is an orally bioavailable oxazolidinone recently approved in China for the treatment of complicated skin and soft tissue infections. In a phase 1 trial, no hematological adverse events were observed with CZD administered at 800 mg twice daily for 28 days (*n* = 10), whereas 2 out of 10 subjects receiving LZD 600 mg twice daily developed hematological adverse events (7, 10). When the same dosing regimens were compared over 7–14 days in a phase 3 trial, CZD was associated with significantly fewer hematological adverse events than LZD (12). An ongoing phase 3 trial will compare the safety and efficacy of CZD and LZD administered for 14–28 days in the treatment of diabetic foot infection (ClinicalTrials.gov Identifier: NCT05369052). Although LZD is commonly used at 600 mg once daily for the treatment of TB, adverse effects still require vigilant safety monitoring and frequently limit the dose and duration of LZD use. Based on the clinical studies performed to date, CZD could represent a better oxazolidinone for TB treatment if it is at least as effective as LZD at exposures that are safer. However, it should be recognized that the potential risk of longer durations of CZD to cause peripheral neuropathy, which typically requires more than 28 days of LZD treatment to become manifest, cannot be adequately assessed from these clinical trials.

As CZD demonstrated potency comparable to LZD against *M. tuberculosis in vitro* and *in vivo*, including similar efficacy to LZD when tested in an acute mouse infection model (9), we investigated the replacement of LZD with CZD in the short-course oral regimen of BDQ, PMD, and LZD. All tested CZD doses showed equivalent efficacy when compared to the same dose of LZD. Available data suggest that CZD and LZD produce similar exposures in mice at the same dose, as measured by the area under the plasma concentration-time curve (AUC) after oral administration. For example, a single oral dose of CZD 90 mg/kg in mice resulted in an average AUC_0–24 h_ of 200.8 µg h/mL (13), comparable to an average AUC_0–24 h_ of 243.5 µg h/mL after a single oral dose of LZD 100 mg/kg (2). Assuming similar exposures of CZD and LZD were achieved at the same doses in our study, our results indicate that CZD may be a suitable candidate to replace LZD in RR-TB treatment, including regimens with BDQ and PMD, if CZD is safer in humans at exposures as high or higher than efficacious LZD exposures.

Studies against *Staphylococcus aureus* showed extremely low frequencies of spontaneous resistance to CZD and LZD between 1 × 10^−12^ and 1 × 10^−13^ (14), with resistance caused by mutations in the 23S rRNA and *rplC* genes. Mutations in these genes are known to confer LZD resistance in *M. tuberculosis* at a frequency of 1 × 10^−8^ (15). The spontaneous frequency of resistance to CZD in *M. tuberculosis* was unexpectedly higher than 10^−6^, or roughly 100 times higher than we observed for LZD. Whole-genome sequence analysis of CZD-selected mutants indicated that the higher frequency of spontaneous resistance was likely driven by mutations in *mce3R*. These results now independently confirm the recently published results from Pi et al. (16), which demonstrated that a variety of loss-of-function mutations in *mce3R* confer resistance to CZD, likely through de-repression of a drug-modifying flavin-dependent mono-oxygenase encoded by *Rv1936*. Consistent with the observed difference in the frequency of spontaneous resistance between CZD and LZD, the CZD-resistant *mce3R* mutant was not cross-resistant to LZD. This finding, also noted by Pi et al. (16), suggests the intriguing possibility that should CZD prove safe and effective enough to replace LZD as the oxazolidinone of choice in TB regimens, and the emergence of resistance to CZD *via* the more frequent *mce3R* mutation would not obviate the future utility of LZD in a re-treatment regimen. Nevertheless, the higher spontaneous frequency of resistance observed for CZD compared to LZD would theoretically place a greater demand on companion drugs to prevent the selection of CZD-resistant mutants during combination therapy.

One limitation of the present study is that it relies on lung CFU counts after 4 weeks of treatment as the sole endpoint for assessing efficacy. Longer durations of treatment and assessment of durable cures after treatment are often used to confirm the sterilizing activity of new drugs and regimens. However, in the present case of replacing a single drug (LZD) within a well-studied regimen (BDQ-PMD-LZD) with a member of the same drug class (CZD), we believe that a relapse study is not necessary to judge whether CZD warrants further clinical evaluation to judge its potential to replace LZD. Previous work in this mouse model has demonstrated a reproducible contribution of LZD to bactericidal and sterilizing activities; and other oxazolidinones (e.g., sutezolid, TBI-223) that increase the bactericidal activity when added to BPa also increase the sterilizing activity of the regimen (2, 17, 18). Therefore, we feel that the results presented here are sufficient to conclude that CZD and LZD may have similar exposure-dependent activity in the clinic. Another limitation of the present study is that it did not include toxicity assessments. Like LZD, CZD inhibits mammalian mitochondrial protein synthesis in a concentration-dependent fashion. This mechanism is responsible for the exposure-dependent hematological and neuropathic toxicity of LZD. Although oxazolidinone toxicity attributable to inhibition of mitochondrial protein synthesis can be measured in mice (19), the model has not been sufficiently validated for this purpose.

In conclusion, CZD is a novel oxazolidinone that is as efficacious as LZD at similar doses in murine models of TB and could prove to be a safer alternative to LZD for TB treatment.

## MATERIALS AND METHODS

### Mycobacterial strains

*M. tuberculosis* H37Rv (ATCC 27294) was passaged in mice, confirmed to be virulent in mice, and stored in 1 mL aliquots at −80° C. Prior to infection, one aliquot of the stored culture was thawed and sub-cultured in Middlebrook 7H9 broth supplemented with 10% (vol/vol) oleic acid-albumin-dextrose-catalase (OADC) enrichment (Becton-Dickinson) and 0.05% (vol/vol) Tween 80 (Fisher Scientific). This inoculum was used for aerosol infection when the optical density at 600 nm was approximately 1.0.

### Antimicrobials

CZD and BDQ were provided by MicuRx Pharmaceuticals and Janssen Pharmaceuticals, respectively. LZD and PMD were provided by the Global Alliance for Tuberculosis Drug Development (New York, NY). Dosing formulations were prepared and maintained as previously described for PMD (20, 21), BDQ, and LZD (22). CZD was prepared in 0.5% carboxymethylcellulose sodium (CMC-Na). All drugs were administered once daily by gavage, 5 days per week; when given in combination, BDQ and PMD were given one after another, and LZD or CZD was given 4 hours later.

### MIC determination

*M. tuberculosis* H37Rv was grown in Middlebrook 7H9 broth supplemented with 10% OADC (BD), 0.5% glycerol, and 0.05% Tween. CZD and LZD stock solutions were prepared in 100% dimethylsulfoxide. Serial twofold dilutions of each stock solution were made with the same 7H9 media but without Tween 80 to yield final assay concentrations ranging from 0.06 to 64 µg/mL. Drug-free tubes served as growth controls. Each polystyrene tube containing 2.4 mL of twofold diluted drug solution was inoculated with 100 µL of *M. tuberculosis* culture adjusted to 0.1 OD at 600 nm to give a final bacterial concentration of approximately 10^5^ CFU mL^−1^. Initial assessment was made after 1 week of incubation, followed by a final assessment at 2 weeks. The MIC was defined as the lowest concentration that inhibited visible bacterial growth after 14 days of incubation at 37°C.

### Mouse infection model

Female BALB/c mice (Charles River, Wilmington, MA) aged 4 to 6 weeks were infected to implant 3.5–4 log_10_ CFU of *M. tuberculosis* H37Rv by the aerosol route using an inhalation exposure system (Glas-col, Inc., Terre Haute, IN). Two mice were sacrificed one day after infection (D-13) to determine the number of CFU implanted in the lungs and five more were sacrificed on the day of treatment initiation (D0) to determine the pre-treatment CFU count.

### Drug treatment

Treatment started 2 weeks after infection (D0) with five mice randomized to each treatment group. The use of five mice per group for CFU counts provides greater than 80% power to detect differences of at least 0.5 log_10_ in group means based on typical variance in the model. Test mice received CZD at 50, 100, or 200 mg/kg, alone or in combination with BDQ at 25 mg/kg and PMD at 100 mg/kg. Control mice received the active comparator LZD at 50 or 100 mg/kg, alone or in combination with BDQ-PMD, or BDQ-PMD alone. All treated mice were sacrificed after 4 weeks of treatment to determine the efficacy of treatment. Lung CFU counts were determined by plating 0.5 mL aliquots of serially diluted lung homogenates on Middlebrook 7H11 selective plates containing 0.4% activated charcoal to minimize the effects of drug carryover (22). All procedures involving animals were approved by the Animal Care and Use Committee of Johns Hopkins University.

### Statistical analysis

Mean lung CFU counts were compared using one-way ANOVA with Dunnett’s post-test (GraphPad Prism).

### Selection and characterization of drug-resistant mutants

A total inoculum of between 10^8^ and 10^9^ CFU was plated on 7H11 agar plates containing CZD at 3 or 10 µg/mL or LZD at 1.5 µg/mL. After 4 weeks of incubation, individual colonies growing on drug-containing plates were selected for confirmatory MIC testing. Genomic DNA from colonies selected on CZD was subjected to whole-genome sequencing at the Johns Hopkins University Next Generation Sequencing Center. Samples were prepared using the TruSeq DNA kit. Sequencing was performed on an Illumina HS2500. Alignments were done by bwa0.7.7 and data aligned to H37Rv genome NC_000962.3. Samtools1.2.1 (mpileup) was used to call variants. snpEFF4.1 was used to annotate the variant calls. Genomic DNA from colonies selected on LZD-containing plates was subjected to PCR to amplify the *rplC* and *rrl* genes known to confer LZD resistance in *M. tuberculosis*, using previously described methods (23).

## Data Availability

Sequencing data have been deposited in the Sequence Read Archive (SRA) repository, BioProject ID: PRJNA1010226.
